# A Spongy Appetite: A Case of Pica

**DOI:** 10.7759/cureus.66399

**Published:** 2024-08-07

**Authors:** Inês Miranda Paulo, Carla Marques, Mariana Serra, Carlota Âmbar Botelho, Ana Alcoforado, Catarina M Pires

**Affiliations:** 1 Family Medicine, Family Health Unit Travessa da Saúde, Local Health Unit São José, Lisbon, PRT; 2 General Practice, Family Health Unit Travessa da Saúde, Local Health Unit São José, Lisbon, PRT

**Keywords:** non-food substances, iron deficiency, anaemia, eating disorders, pica

## Abstract

Pica is an eating disorder defined as the compulsive and repeated ingestion of substances that have no nutritional value for at least one month. This condition may be hard to diagnose without complications, as a high degree of suspicion is needed.

The subject in this case was a teenager who presented with asthenia and unspecific abdominal pain. The etiological workup showed no abnormalities other than mild anemia and iron and folate deficiencies. After a thorough anamnesis, the patient's mother mentioned sporadic ingestion of synthetic mattress foam since childhood, which had become more frequent in the previous year. With this key information, it was possible to establish a diagnosis before serious complications occurred and thus help the patient get the necessary assistance by referring them to pediatrics, nutrition, and child and adolescent psychiatry consultations.

This case report highlights the importance of a detailed anamnesis, particularly when dealing with unspecific symptoms, exploring the possibility of disorders that are rarely thought of, such as pica. It also recaps how important it is to address sensitive topics like eating disorders and create an open environment with no judgment, as these attitudes are crucial to ensuring the correct diagnosis and providing the best care for patients.

## Introduction

According to the Diagnostic and Statistical Manual of Mental Disorders, fifth edition (DSM-5), pica is defined as the “persistent eating of non-nutritive, non-food substances for at least one month”. Furthermore, these eating behaviors should not be part of a socially or culturally accepted practice nor appropriate to the development of the individual. As such, pica cannot be diagnosed in children under two years of age [1].

The prevalence of pica is difficult to establish because of patients’ reluctance to admit to abnormal cravings and ingestion [2]. Few cases are reported and occur most commonly in children and pregnant women [3,4]. The latest data estimated its prevalence up to 27.8%, which is higher in Africa than in other continents [5]. However, the incidence is the same for both genders [6]. Ingested substances vary with age and availability and might include ice (pagophagia), soil (geophagia), raw starches (amylophagia), paper (xylophagia), and hair (trichophagia), among others [4,6].

Pica usually occurs as an isolated disorder, but it may coexist with other mental disorders, such as schizophrenia, autism spectrum disorders, and obsessive-compulsive disorders [2,5]. It is also a common eating disorder in patients who are intellectually impaired [4,5]. Other risk factors include child neglect and abuse, malnutrition, oral overstimulation, stress, low socioeconomic status, and epilepsy [5].

It is believed that the etiology of pica is multifactorial, though no direct association has been established [3]. Deficiencies in iron, calcium, zinc, and many vitamins, including vitamins C, D, thiamine, and niacin, have been associated with pica, although no pathophysiological explanation was correctly found [4].

Another hypothesis establishes that these non-nutritive substances offer protection from harmful toxins during vulnerable stages of human cell replication and embryogenesis occurring during childhood and pregnancy. It is suggested that the protection is binding to toxins, consequently decreasing their intestinal absorption [5].

Symptoms may vary according to the patient’s health status and the substance consumed [2]. Gastrointestinal symptoms can range from abdominal pain to perforation, blockage, and ischemia. Vitamin and mineral deficiency are reported to have a bidirectional effect, both resulting from and contributing to pica [2,4]. Manifestation of parasitic infestation or heavy metal exposure may be present [2].

Pica is a clinical diagnosis, and a complete anamnesis is essential to convey specific laboratory investigations [3,5]. For instance, iron deficiency should be sought in pregnant women, and lead poisoning should be discarded in cases involving paint or chalk [5]. Differential diagnoses may include other eating disorders, such as anorexia nervosa, factitious disorder, and personality disorders [5]. These patients should be included in a multidisciplinary intervention, with physicians, dietitians, behavioral therapists, and social workers [5].

Treating pica can be a challenge, as no medication is specifically designed [5]. Nevertheless, successful treatment has been seen in patients due to selective serotonin reuptake inhibitors, atypical neuroleptics, and attention-deficit disorder medication [2]. However, when a deficiency is identified, supplementation should be offered to the patients [5]. Behavioral treatment is the most effective treatment and might be most helpful in patients with mental disorders [2]. Reducing exposure to craving substances, either by limiting access or finding an appropriate substitute with a similar texture or flavor, is also helpful [2,5]. Pica is usually benign but can persist in patients who suffer from other mental illnesses [3].

This article was previously presented as a meeting abstract at the Jornadas Salvador Allende Meeting on November 27, 2023.

## Case presentation

This case involves a 16-year-old girl born in São Tomé and Príncipe, who has been living in Portugal since 2014, except for a temporary return to her home country in 2022 for approximately one year. She lives with her mother and extended family, including her uncles and grandmother.

The Graffar index characterizes the population's socioeconomic status using five criteria: educational level, sources of income, housing comfort, and neighborhood appearance. This scale assesses the household's socioeconomic level, living conditions, and available resources. In this case, a Graffar index assessment indicated a low socioeconomic status. Her medical history includes an insulinoma, for which she underwent a pancreaticoduodenectomy in 2017, and iron deficiency anemia, which was treated successfully with oral iron supplementation from 2017 to 2018 (Table 1). There is no known psychiatric history.

**Table 1:** Analytic values before and after iron supplementation (2017-2018)

	July 2017	June 2018	Reference values
Erythrocytes	4.79 10E6/mm³	4.69 10E6/mm³	4.2-5.4 10E6/mm³
Hemoglobin	8.5 g/dL	12.9 g/dL	12-15.5 g/dL
Hematocrit	29.20%	38.40%	36%-46%
Mean corpuscular volume	61 fL	81.9 fL	80-100 fL
Mean corpuscular hemoglobin	17.7 pg	27.5 pg	27-32 pg
Mean corpuscular hemoglobin concentration	29.1 g/dL	33.6 g/dL	31-36 g/dL
Ferritin	4.5 ng/mL	9.1 ng/mL	10-150 ng/mL
Iron	17 µg/dL	46 µg/dL	50-150 µg/dL

After four years without attending surveillance appointments, the 16-year-old girl presented at her family medicine center with complaints of asthenia and diffuse abdominal pain over the previous year. She reported no changes in bowel movement patterns, urinary or gynecological symptoms, nausea, or vomiting.

During anamnesis, the mother mentioned that the girl had sporadically ingested synthetic foam from mattresses since childhood, with a noticeable increase in this behavior over the past year, possibly linked to increased family stress and their recent relocation to Portugal.

Anthropometric data in January 2023 were as follows: height of 164 cm, weight of 52 kg, and body mass index of 19.3 kg/m^2^. The physical examination revealed slight mucocutaneous pallor, but all other physical findings, including the abdominal examination, were normal.

An analytical study was performed, detecting hypochromic and microcytic anemia related to iron deficiency (hemoglobin levels of 11,6 g/dL) with low iron (serum ferritin of 8.7 ng/mL and serum iron levels of 31 µg/dL) and folate (3.7 ng/mL) levels; other values are given in Table 2. The abdominal ultrasound showed no relevant findings.

**Table 2:** Analytic values before and after iron supplementation in 2023

	January 2023	June 2023	Reference values
Erythrocytes	4.36 10E6/mm³	4.29 10E6/mm³	4.2-5.4 10E6/mm³
Hemoglobin	11.6 g/dL	12 g/dL	12-15.5 g/dL
Hematocrit	35.10%	36.20%	36%-46%
Mean corpuscular volume	81 fL	84 fL	80-100 fL
Mean corpuscular hemoglobin	27 pg	28 pg	27-32 pg
Mean corpuscular hemoglobin concentration	33 g/dL	33 g/dL	31-36 g/dL
Ferritin	8.7 ng/mL	7.2 ng/mL	10-150 ng/mL
Iron	31 µg/dL	189 µg/dL	50-150 µg/dL
Folic acid	3.7 ng/mL	15.3 ng/mL	3-20 ng/mL

Daily supplementation with 150 mg of oral iron and 5 mg of oral folic acid was started, and the patient was referred to pediatrics, nutrition, and child and adolescent psychiatry appointments. A month later, during a follow-up appointment, she reported improvement in asthenia and abdominal pain, with no new episodes of ingesting synthetic foam or other non-nutritive substances. Five months after the initial appointment, an analytical examination revealed improved anemia and higher iron levels (Table 2). At the time of writing this study, the patient was under pediatric monitoring and awaiting an appointment with child psychiatry. To summarize, a timeline of the patient’s events is included in Table 3.

**Table 3:** Timeline of the patients’ relevant life events

Timeline of relevant life events	
2006	Born in São Tomé and Príncipe (STP)
2014 (8-years old)	Moved to Portugal with her grandmother, and her mother stayed in STP.
2017 (11-years old)	Following an episode of loss of conscience and convulsions, the patient went to the emergency room and was diagnosed with insulinoma, for which she underwent a pancreaticoduodenectomy, cholecystectomy, and excision of the antrum of the stomach; iron deficiency anemia, which was treated with oral iron supplementation; *H. pylori*-positive chronic gastritis, for which eradication treatment was instituted.
2019-2022	No medical surveillance. Moved to France for a year in 2020 and later returned to STP for approximately a year in 2021.
February 2023 (16-years old)	Returned to Portugal, complaining about abdominal pain, hematochezia, and asthenia. Sporadic ingestion of mattress foam since childhood was mentioned by the patient’s mother. The analytical study revealed hypochromic and microcytic anemia related to iron and folate deficiencies. The patient started daily iron and folic acid supplementation and was referred to pediatrics, nutrition, and child and adolescent psychiatry appointments.
March 2023 (17-years old)	No new episodes of ingestion of foam or any other non-nutritive substances. Improvement in her asthenia and abdominal pain complaints.
June 2023 (17-years old)	A new analytical study showed correction of the anemia, with increased levels of iron and folic acid.

## Discussion

Pica is an eating disorder with a multifactorial etiology that is not yet completely understood and constitutes a challenge in daily practice, as it involves taking a careful clinical history, with a holistic approach to the patient [2]. This uncommon condition with nonspecific signs and symptoms is often forgotten, particularly in the absence of complications. Numerous substances are associated with pica [4,6], thus requiring a high degree of suspicion for its diagnosis. Furthermore, its diagnosis also requires concern from the family or the patients themselves regarding those symptoms [7].

First, it is important to highlight the role of the family doctor during the patient’s initial contact with healthcare, particularly within the Child and Youth Health surveillance program, implemented by the General Directorate of Health in Portugal. This program schedules consultations at key ages, ensuring close and frequent monitoring throughout the various stages of a child's development. In this context, family doctors are in a privileged position to promote health, provide anticipatory care, and diagnose eating disorders such as pica [8]. In this case, the non-attendance of Child Health consultations for over four years stalled proper follow-up, adequate monitoring of the patient’s health status, thorough investigation of relevant signs and symptoms, and exploration of any family concerns [9]. This led to delayed diagnosis, resulting in late institution of treatment and referral to hospital consultation, ultimately negatively impacting the patient’s health [2,5,10].

Additionally, a thorough anamnesis in routine Child and Youth appointments is essential [8,11]. During these consultations, doctors should always evaluate the patient’s dietary habits, opening the door to identifying possible abnormal behaviors [8]. Even if the parents underestimated these symptoms, the interviews conducted during the appointments should raise suspicion and facilitate the diagnosis of pica.

One of the main risk factors associated with the development of pica is iron deficiency [9]. In this case, the patient had a history of iron deficiency anemia, which had been previously treated with oral iron supplements. However, there was no revaluation or follow-up blood tests, raising doubts about the correct treatment of this condition. It is possible that the iron deficiency was not fully treated, thus contributing to the development of pica [11,12]. Furthermore, pica can occur even in the absence of established anemia as an iron deficiency alone, without repercussion in blood count, and can trigger these symptoms [10,12]. The pathogenesis is still unclear, although it appears unrelated to the degree of iron deficiency [13].

It is also important to highlight the multifactorial nature of pica. No isolated theory can fully explain this condition, making it necessary to evaluate the nutritional, family, and sensory aspects [2]. Evaluating family involvement is crucial, and this patient’s precarious familial situation, characterized by stress, family disorganization, and lack of encouragement for autonomy, is significant. The application of the Graffar index allows assessment of the patient’s socioeconomic situation, which is relevant, as pica has been linked to low socioeconomic status and underdevelopment areas [11]. It is also important to emphasize the sensorial component of this pathology, which translates into enjoying the taste, the texture, or even the smell of the sponge or other items.

In this case, all these diverse factors contributed to the abnormal eating behavior [2]. Due to the lack of follow-up, the patient was evaluated in an urgent consultation for abdominal pain and not a routine appointment. This was an unsuitable setting for addressing such a condition, as these nonspecific symptoms could have been undervalued or overlooked in urgent care, highlighting the challenges in diagnosing pica [7,10]. A scheduled appointment would have been a more appropriate environment to thoroughly explore the patient’s complaints and address the parent’s concerns. The management included prompt oral iron supplementation [12], behavioral and dietary therapy, and follow-up hospital appointments, resulting in a positive outcome for the patient.

As the signs and symptoms are usually underrated and underreported, diagnosis is often made when complications arise, such as anemia, lead poisoning/toxicity, intestinal obstructions, malnutrition, or other metabolic conditions [10].

In conclusion, family doctors must remain vigilant regarding clinical history elements that indicate this behavior, addressing the ingestion of unusual substances directly and objectively, for a better diagnostic capacity and possible therapeutic guidance. This eating disorder is usually treatable, and symptoms usually relapse when supplementation is introduced. However, pica requires a multidisciplinary approach, involving nutritional supplementation, behavioral therapy, and dietary education for effective management.

## Conclusions

Pica is an uncommon condition with unspecific signs and symptoms, making its diagnosis truly challenging in the absence of complications, a high degree of suspicion, or communicated concerns from the patient or those close to the patient. Treating this condition also constitutes a challenge, as no specific medication is available. Management requires a multidisciplinary approach that includes nutritional supplementation, behavioral therapy, and dietary education.

Family doctors are in a privileged position to diagnose eating disorders such as pica due to their holistic approach to patient care. They should closely monitor the child’s development stages, including dietary habits, and possess insights into family dynamics, thus recognizing potential risk factors.
